# IL-12p40 Deficiency Leads to Uncontrolled *Trypanosoma cruzi* Dissemination in the Spinal Cord Resulting in Neuronal Death and Motor Dysfunction

**DOI:** 10.1371/journal.pone.0049022

**Published:** 2012-11-12

**Authors:** André Luis Bombeiro, Lígia Antunes Gonçalves, Carlos Penha-Gonçalves, Claudio Romero Farias Marinho, Maria Regina D’Império Lima, Gerson Chadi, José Maria Álvarez

**Affiliations:** 1 Department of Immunology, Biomedical Sciences Institute, University of São Paulo, São Paulo, São Paulo, Brazil; 2 Neuroregeneration Center, Department of Neurology, University of São Paulo School of Medicine, São Paulo, São Paulo, Brazil; 3 Instituto Gulbenkian de Ciência, Oeiras, Portugal; 4 Department of Parasitology, Biomedical Sciences Institute, University of São Paulo, São Paulo, São Paulo, Brazil; State University of Campinas, Brazil

## Abstract

Chagas’ disease is a protozoosis caused by *Trypanosoma cruzi* that frequently shows severe chronic clinical complications of the heart or digestive system. Neurological disorders due to *T. cruzi* infection are also described in children and immunosuppressed hosts. We have previously reported that IL-12p40 knockout (KO) mice infected with the *T. cruzi* strain Sylvio X10/4 develop spinal cord neurodegenerative disease. Here, we further characterized neuropathology, parasite burden and inflammatory component associated to the fatal neurological disorder occurring in this mouse model. Forelimb paralysis in infected IL-12p40KO mice was associated with 60% (p<0.05) decrease in spinal cord neuronal density, glutamate accumulation (153%, p<0.05) and strong demyelization in lesion areas, mostly in those showing heavy protein nitrosylation, all denoting a neurotoxic degenerative profile. Quantification of *T. cruzi* 18S rRNA showed that parasite burden was controlled in the spinal cord of WT mice, decreasing from the fifth week after infection, but progressive parasite dissemination was observed in IL-12p40KO cords concurrent with significant accumulation of the astrocytic marker GFAP (317.0%, p<0.01) and 8-fold increase in macrophages/microglia (p<0.01), 36.3% (p<0.01) of which were infected. Similarly, mRNA levels for CD3, TNF-α, IFN-γ, iNOS, IL-10 and arginase I declined in WT spinal cords about the fourth or fifth week after infection, but kept increasing in IL-12p40KO mice. Interestingly, compared to WT tissue, lower mRNA levels for IFN-γ were observed in the IL-12p40KO spinal cords up to the fourth week of infection. Together the data suggest that impairments of parasite clearance mechanisms in IL-12p40KO mice elicit prolonged spinal cord inflammation that in turn leads to irreversible neurodegenerative lesions.

## Introduction

Chagas’ disease is an endemic Latin America illness caused by the protozoan *Trypanosoma cruzi*. During the acute phase of the infection, symptomatic patients present fever, enlargement of the liver, lymph nodes and spleen and intense inflammatory processes associated with tissue parasitism, while during the chronic phase of the disease, they can develop cardiomyopathy and/or digestive megasyndromes [1], [2], [3] or, not less frequent, remain asymptomatic for life [2].

*T. cruzi* infection induces an innate immunity entailing production of IL-12 and TNFα by dendritic cells/macrophages and IFN-γ by NK cells. Subsequently, activation of acquired immunity occurs, with IL-12-stimulated-differentiation and expansion of TH1 CD4^+^ cells, specific antibodies response and generation of CD8^+^ cytotoxic cells that destroy parasitized cells [3]. The production of IFN-γ by CD4^+^ and CD8^+^ T cells enhances the effector activity of phagocytes (production of reactive nitrogen and oxygen intermediates – RNIs and ROIs – and GTPAses), a machinery that becomes highly effective when working in conjunction with opsonization induced by specific IgG. Nevertheless, *T. cruzi* parasites frequently manage to evade the immune system response [4], [5], establishing a chronic infection.

Although *T. cruzi* can be found in the spinal fluid early in the infection [6], it rarely causes damage to the central nervous system (CNS), unless the patient is immunosuppressed, as reported in cases of anti-allograft rejection drug treatment or HIV infection. In such cases, patients develop meningoencephalitis, intracranial hypertension, neurological deficits and focal CNS lesions owing to local infection reactivation [7], [8]. Parasite nests and infiltrating leukocytes in the brain of immunocompetent mice have also been reported [9], a process that is notably enhanced in IL-12/IL-23- (IL-12p40-) and IFN-γ-deficient mice [9], [10], [11]. Recently, we showed that *T. cruzi* X10/4 strain infected IL-12p40-knockout (IL-12p40KO) mice developed motor impairments, culminant in death [11]. The neurological syndrome was concurrent with an intense inflammation in the spinal cord, associated with high local production of IFN-γ and nitric oxide (NO), but curiously with no efficient control of the parasitic load [11]. Conversely, paired *T. cruzi* infected immunocompetent (WT) mice never displayed motor alterations.

In response to a damage in the nervous tissue, glial cells commonly release proinflammatory mediators, free radicals and proteases as an effort to reestablish tissue homeostasis [12], [13]. Nevertheless, the overproduction of those substances evoked by persistent antigenic stimulation results in neuronal death [14], [15]. Additionally, CNS-intrinsic factors such as the neurotransmitter glutamate can also account for the neurodegenerative process when they are continuously released [16].

Here, we compared the kinetics of parasite load and locally-evoked immune response in the spinal cord of IL-12p40KO and WT mice that were infected by *T. cruzi* Sylvio X10/4 parasites. In addition, we performed a detailed evaluation of the motor dysfunctions following infection of IL-12p40KO mice and analyzed the involvement of neurons, microglia/macrophages and astrocytes in the neurodegenerative process.

## Materials and Methods

### Mice, Parasites and Infection

Eight- to ten-week-old female C57BL/6 wild-type (WT) mice and IL-12/IL-23-deficient (IL-12p40KO) mice [17] in the C57BL/6 background were bred under pathogen-free conditions in our isogenic mouse facilities, in the Biomedical Sciences Institute of the University of São Paulo. They were kept under controlled temperature and humidity in a light and dark cycle of 12 h each and fed *ad libitum*. Mice were intraperitoneally infected with 10^5^ trypomastigote forms of *T. cruzi* Sylvio X10/4 clone [18] that were obtained from infected LLCMK2 cells. Food and water were placed on the bottom of the cage when IL-12p40KO mice developed motor deficits. All protocols were approved by the Health Animal Committee of the Biomedical Sciences Institute of the University of São Paulo (permit number 140), which guidelines for animal use and care were based on the standards established by the Animal Experimentation Brazilian College (COBEA) and the Canadian Council of Animal Care.

### Behavioral Evaluation

Mice were weighed and evaluated for general and neurological conditions three times a week until they started presenting signs of paralysis. Afterwards, they were monitored almost daily, being employed the following behavioral analyses. (a) Spontaneous motor activity was assessed by placing the mouse in an open field and evaluating spontaneous movements by eye. A clinical score based on Becher *et al*. [19] was developed to quantify the motor activity (Table 1). (b) The inclined plane test, based on Rivilin and Tator [20], was used to assess motor function, wherein each mouse was placed on a rubber mat plane with its body kept in parallel to the plane’s longitudinal axis and the head oriented downwards while the plane was inclined. The grade corresponding to the maximum angle that the animal could maintain its position for 5 seconds without falling was assessed, a mark that in healthy mice corresponds to 50 to 60 degrees.

**Table 1:** Scale of motor impairments based on spontaneous motor activity.*

Score	Signs
0	No detectable signs of paralysis
1	Loss of muscular tonus in the tail distal region
2	Loss of muscular tonus in the tail proximal region
3	One limp hind limb (distal portion)
4	Proximal and/or distal paralysis of one hind limb or distal paralysis of both hind limbs
5	Bilateral distal and proximal paralysis of both hind limbs
6	Spine alteration (visible humpback)
7	Proximal and/or distal paralysis of one forelimb or distal paralysis of both forelimbs
8	Bilateral proximal and distal paralysis of both forelimbs

* Modified from Becher *et al*. [19].

### Tissue Preparation, Sectioning and Sampling

Mice were euthanized with overdoses of ketamine and xylazine (Vetbrands) and transcardially perfused with 10 mL of isotonic saline at room temperature, followed by 50 mL of a fixative solution consisting of 4% paraformaldehyde (w/v, Merck) in 0.1 M phosphate buffer (PBS) (pH 6.9) for 6 min. For stereological and microdensitometric/morphometric image analysis, the entire spinal cord was removed, post-fixed in a 4% paraformaldehyde solution at 4°C for 90 min and rinsed for 48 h at 4°C in a 10% sucrose (Merck) solution dissolved in 0.1 M PBS (pH 7.4). The harvested spinal cords were separated into three segments (cervical, thoracic and lumbar regions), which were oriented along the rostrocaudal axis, immersed in a tissue freezing medium (Jung) and frozen in dry-ice-cooled isopentane (Sigma) at -45°C. Transversal sections (14-µm thick) were obtained using a cryostat. The three segments were systematically cut so that the entire spinal cord was sampled throughout nine distinct points. For each IL-12p40KO spinal cord section, one paired WT section that was obtained from the same level was placed beside it. Glass slides were stored at -70°C until use. For histopathological analysis, the upper lumbar intumescences of the spinal cord were fixed overnight in formalin 4% (Merck), followed by alcohol 70%, embedded in paraffin, sectioned and stained with haematoxylin-eosin (HE). Under a 400× magnitude, the entire spinal cord area of 3 non-adjacent sections was investigated.

### Immunohistochemical Procedures

To use the single-color immunoperoxidase method for single antigen detection, the sections were washed 2× for 10 min in 0.1 M PBS (pH 7.4), incubated with a 5% fat-free milk solution in PBS for 1 h at room temperature, washed 2× for 10 min in PBS and incubated with 0.05% H_2_O_2_ in PBS (v/v, Sigma) for 30 min to quench endogenous peroxidase. Sections were washed again 2× for 10 min in PBS and incubated overnight at 4°C with one of the following epitope-specific antibodies that had been diluted in PBS containing 0.5% Triton X-100 (Sigma) and 1% bovine serum albumin (Sigma): rabbit anti-neurofilament-200 (NF-200, Sigma) at a 1∶600 dilution, rabbit anti-glial fibrillary acid protein (GFAP, Dako) at a 1∶1,200 dilution, rabbit anti-nitrotyrosine (Sigma) at a 1∶500 dilution, rabbit anti-glutamate (Sigma) at a 1∶1,000 dilution, rabbit anti-arginase I (Santa Cruz Biotechnology) at a 1∶500 dilution and biotin rat anti-mouse CD11b (BD Pharmingen) at a 1∶600 dilution. After two 10 min washes in PBS, sections (except those already incubated with anti-CD11b) were incubated with biotinylated goat anti-rabbit IgG (Vector) that had been diluted 1∶250 as described above for 1 h at room temperature. Thereafter, all sections were rinsed 2× for 10 min in PBS and incubated with an avidin and biotin-peroxidase complex (both 1∶125, Vector) for 45 min at room temperature, and again they were washed 2× 10 min in PBS and 1× 10 min in 0.05 M tris buffer (pH 7.4; Sigma). Immunoreactivity was visualized using 3–3′-diaminobenzidine tetrahydrochloride (DAB, 0.03% w/v; Sigma) as a chromogen and H_2_O_2_ (0.05%, v/v; Sigma), wherein both were diluted in Tris solution. The reaction was interrupted with Tris solution when the darkest elements in the sections were below saturation, which was generally after three minutes.

Two-color immunoperoxidase methods were used to simultaneously detect *T. cruzi*/NF200, *T. cruzi*/GFAP, *T. cruzi*/CD11b, myelin basic protein (MBP)/NF200 and MBP/nitrotyrosine. After following conventional one-color immunoperoxidase procedures for NF-200, GFAP, CD11b and nitrotyrosine detection, as described above, the sections were washed 3× for 10 min in PBS (pH 7.4), followed by incubation with 5% H_2_O_2_ in PBS (v/v; Sigma) for 30 min so as to quench the biotin-peroxidase. The sections were washed again 4× for 10 min in PBS and incubated for 96 h at 4°C with 1∶100 diluted rabbit anti-MBP antibody (DAKO) or with a rat polyclonal anti-*T. cruzi* Sylvio X10/4 serum (1∶70), which was obtained from a chronically infected rat, and absorbed into a mixture of mouse spinal cord tissue and spleen cells so as to remove nonspecific antibodies. Anti-*T. cruzi* serum and anti-MBP antibody were diluted as described above, and the dilution and/or incubation procedures for secondary antibody (biotinylated goat anti-rat Igs, BD Pharmingen or biotinylated goat anti-rabbit IgG; Vector) and avidin and biotinylated peroxidase were the same as for one-color immunoperoxidase. Immunoreactivity was visualized using 4-chloro-naphthol as a chromogen (0.05%, w/v; Sigma) and H_2_O_2_ (0.03%, v/v; Sigma), wherein both were diluted in Tris (pH 7.6). The reaction was interrupted using Tris solution after approximately 10 minutes.

### Stereological Analysis

Spinal cord sections that had been obtained from WT (n = 3) and paralyzed IL-12p40KO mice (n = 3) around seven weeks after infection were sampled as above, immunolabeled for NF-200 (for neurons) or CD11b (for macrophages/microglia) and counterstained with Giemsa. Thereafter, the material was submitted to the optical fractionator method as to estimate the total number of cell body profiles in the entire spinal cord regardless of the presence of gray and white matter and including lesion sites. Two independent procedures were performed to estimate the number of infected and non-infected macrophage/microglia, wherein the sum of both values corresponded to the total number of cells. Of note, all CD11b immunoreactive cells with polymorphonuclear-shaped nuclei were discarded from this analysis. Every 210^th^ section was systematically sampled (*f*1 = 210) and analyzed using a computer-assisted stereological toolbox (CAST) system, as previously described [21]. Briefly, the microscope was interfaced with a computer through a color video camera. The GRID software package was used to control the motorized *X*-*Y* stage, and a microcator, also linked to the microscope, monitored movements in the vertical direction (*Z*). The border of the entire section was outlined, the step rates were entered (500 µm in the *X* and *Y* directions) and the program created a series of uniform sample fields throughout the section. A 100× oil-immersion objective was used to count the cells (*Q* ^−^) in counting frames (2311 µm^2^, *a*_frame_) that were created by the software. From the step rates, it was possible to calculate the second sampled fraction (*f* 2) based on the equation [(*X* step-length ⋅ *Y* step-length)/*a* frame]. The sampling volume (dissector) on the *Z*-axis extended 8- µm deep (height of the dissector) after excluding portions of the section that were close to the slide and cover slip. The total thickness of the section was also measured, giving the third sampling fraction (*f* 3): height of the section/height of the dissector. After counting all cells, we estimated the total cell numbers in the sampled region of the spinal cord, from the cervical to sacral level, according to the following formula: (*N*_total_ = Σ*Q* ^−^·*f*1·*f*2·*f*3). The coefficient of error (CE) for this procedure was obtained as previously described [22]. The total volume of the spinal cord was determined in the sampled sections by multiplying the cross-sectional areas of the delineated sections by the distance between them.

### Semi-quantitative Morphometric Image Analysis

Glial fibrillary acid protein (GFAP, an astrocyte marker) and glutamate immunoreactivities were determined on all sampled spinal cord sections of WT (n = 3) and paralyzed IL-12p40KO mice (n = 3) around seven weeks after infection via the microdensitometric/morphometric image analysis in a Kontron-Zeiss KS400 image analyzer. For WT mice, whose spinal cord tissue was preserved, measurement fields (44.1×10^−3^ mm^2^ in size) were sampled in the anterior and posterior horns. For IL-12p40KO mice, we chose fields (of the same size) that bordered infiltrating cell loci or, in the absence of inflammatory foci, in the anterior and posterior horns. These procedures have been previously described [23]. Briefly, a video camera was used to acquire images from the microscope (x40 objective). After shading correction, a discrimination procedure was performed as follows: the mean gray values (MGV) and standard error of the mean (SEM) were measured in the above-described fields and in the corresponding spinal cord areas that were devoid of specific labeling (background). A specific (sp) MGV was defined as the difference between the background MGV and the MGV of the discriminated profiles. The glass value was kept constant at 200 MGV. The procedure was repeated for each section so as to correct each specific labeling measurement for the background. All of the immunoreactive profile areas were differentiated, including measurements of the cytoplasm and processes (when present and labeled), which reflects the state of the reactive cells and the amount of cells that are involved in a specific event.

### Relative Quantification of mRNA by Real-time RT-PCR

After transcardiac perfusion with 10 mL isotonic saline at room temperature, anesthetized *T. cruzi*-infected WT (n = 4 per time point) and IL-12p40KO mice (n = 4 per time point), as well as uninfected WT and IL-12p40KO mice (n = 4) had their spinal cords removed and the lower lumbar intumescences immediately dry-ice frozen and kept in liquid nitrogen. Total RNA was isolated from the samples using TriZol reagent (Invitrogen) according to the manufacturer’s protocol, and the concentration of RNA in each sample was estimated by using a fluorescent RNA quantification kit (Quant-it, Invitrogen). Samples were then incubated with DNAse to digest contaminating DNA and the reverse transcription was performed with 2 µg of total RNA, utilizing random primer oligonucleotides (Invitrogen). Amplification was performed with Real-Time PCR System (ABI Prism 7900HT, Applied Biosystems). *Trypanosoma cruzi 18S rRNA*, CD3 (*Cd3e*), TNF-α (*Tnf*), IFN-γ (*Ifng*) and HPRT expression was quantified using Power SYBR Green Master Mix (ABI), the primer sequence for *T. cruzi* 18S rRNA (FW: TTGAATTGAGGGCCTCTAAGG; RW: AAAGGTACCACTCCCGTGTTT), CD3 (FW: TCTCGGAAGTCGAGGACAGT; RW: ATCAGCAAGCCCAGAGTGAT), TNF-α (FW: AATGGCCTCCCTCTCATCAGTT; RW: CCACTTGGTGGTTTGCTACGA), IFN-γ (FW: CACACTGCATCTTGGCTTTG; RW: TCTGGCTCTGCAGGATTTTC) and HPRT (FW: GTGGACTTCATTCGCCTCAAG; RW: TCTCCACCGATAACTTTGATTTCA). For iNOS (*Nos2*), IL-10 (*Il10*) and arginase I (*Arg I*) expression was quantified using TaqMan Gene Expression Assays from ABI (Mm00440502 m1, Mm00439614 m1 and Mm00474988 m1, respectively) with TaqMan Gene Expression Master Mix (ABI). The gene expression reactions were performed according to the manufacturers’ instructions for Real-Time PCR System. All samples were analyzed in duplicate and relative mRNA levels were obtained by normalizing the target gene to an endogenous reference (Actb (Mouse ACTB Endogenous Control, ABI) and Gapdh (Mouse GAPDH Endogenous Control, ABI) were used in multiplex PCR, the former with iNOS and arginase I and the latter with IL-10, or HPRT for the others) and then relative to a calibrator sample (uninfected mice), by applying the 2^−ΔΔCt^ formula, being mRNA levels expressed as *n*-fold difference relative to the calibrator. For *T. cruzi* 18S rRNA absolute quantification, sample values were fitted into a standard curve, constructed from a pattern sample with known number of copies of *T. cruzi* 18S rRNA.

### Statistical Analysis

Data were analyzed by using two-way Analysis of Variance (ANOVA) when comparing paired WT and IL-12p40KO lineages through a time course or when submitted to different treatments. For comparing two or more groups belonging to the same lineage, one-way ANOVA was carried out. Following ANOVA, Bonferroni post-tests were employed. Unpaired *t*-tests were used to analyze data from image and stereological analyses. Data are expressed as mean ± standard error (SE). Probability (*P*) values <0.05 were considered significant. All tests were performed by PRISM 4 software (Graph-Pad Software).

## Results

### Neurological Evaluation

The *T. cruzi* Sylvio X10/4 clone is a low virulence strain that has been shown not to cause patent parasitemia in WT mice [10], [24]. Similarly, our analysis indicated a lack of parasitemia in both infected WT and IL-12p40KO mice over the experimental period. Body weight decreased in the IL-12p40KO group later in the infection, whereas it increased in WT mice despite small oscillations (data not shown).

**Figure 1:**
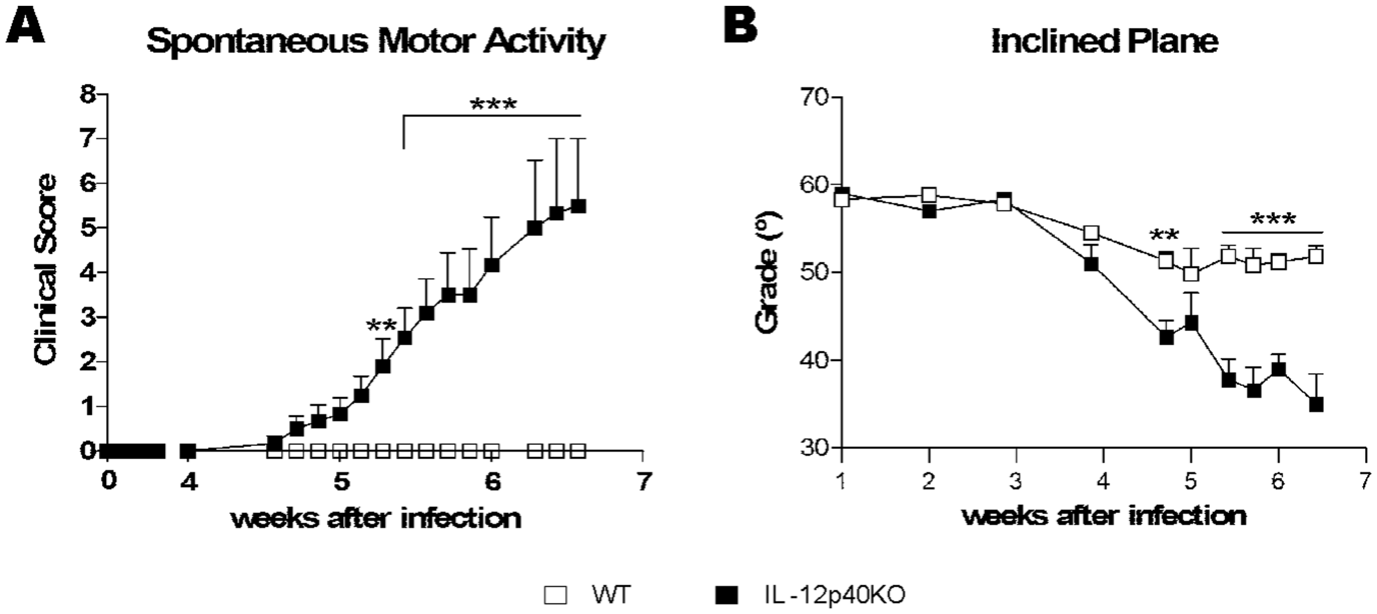
Neurological scoring after *T. cruzi* infection. A) *T. cruzi* Sylvio X10/4-infected WT (n = 14) and IL-12p40KO (n = 14) mice were placed in an open field, evaluated by eye for spontaneous motor activity in a timed course and scored according to Table 1 (from 0, no paralysis, to 8, complete forelimb paralysis). While WT mice presented no sign of paralysis, IL-12p40KO mice displayed an ascending paralysis from the tail to the forelimbs. B) Infected mice were submitted to the inclined plane test (WT, n = 6; IL-12p40KO, n = 8) to assess motor function, wherein healthy mice are able to stay on a plane with a slope varying from 50 to 60 degrees. Data show that IL-12p40KO mice manifested impaired motor function, whereas WT mice did not. Mean ± SEM. **p<0.01; ***p<0.001 when comparing paired WT and IL-12p40KO groups according to Bonferroni post-tests.

Our previous work identified a deficit in the spontaneous motor activity in *T. cruzi*-infected IL-12p40KO mice [10], [11]. To quantify motility disability we applied a clinical score based on the report by Becher and colleagues [19], comprising the most conspicuous signs of the motor dysfunction (Table 1). According to the spontaneous motor activity analysis, paralysis had an ascending progression in IL-12p40KO mice, first affecting motility of distal tail regions, followed by impairment of the total tail, hind limbs, spine and, finally, forelimbs. Contrasting to knockout mice, WT mice displayed no signs of paralysis (Figure 1A). Complementing these data, the inclined plane test showed that WT mice managed to stay on the plane with a slope that varied from 50 to 60 degrees, whereas IL-12p40KO mice were not able to reach such values after 4 weeks of infection (Figure 1B). In addition, the unbiased automatic recording of spontaneous motor activity (Protocol S1) showed that infected IL-12p40KO mice behaved differently in regard to resting stops, which were fewer and of longer duration (Figure S1A and B) and to large movements, which were fewer and briefer (Figure S1C and D). No motility recovery was ever observed and the mice succumbed after six weeks post-infection. This analysis demonstrates that *T. cruzi* infection in IL-12p40KO mice leads to an irreversible moto-neurological disorder with a fatal outcome.

### Neuronal Death and Glial Response after *T. cruzi* Infection

**Figure 2:**
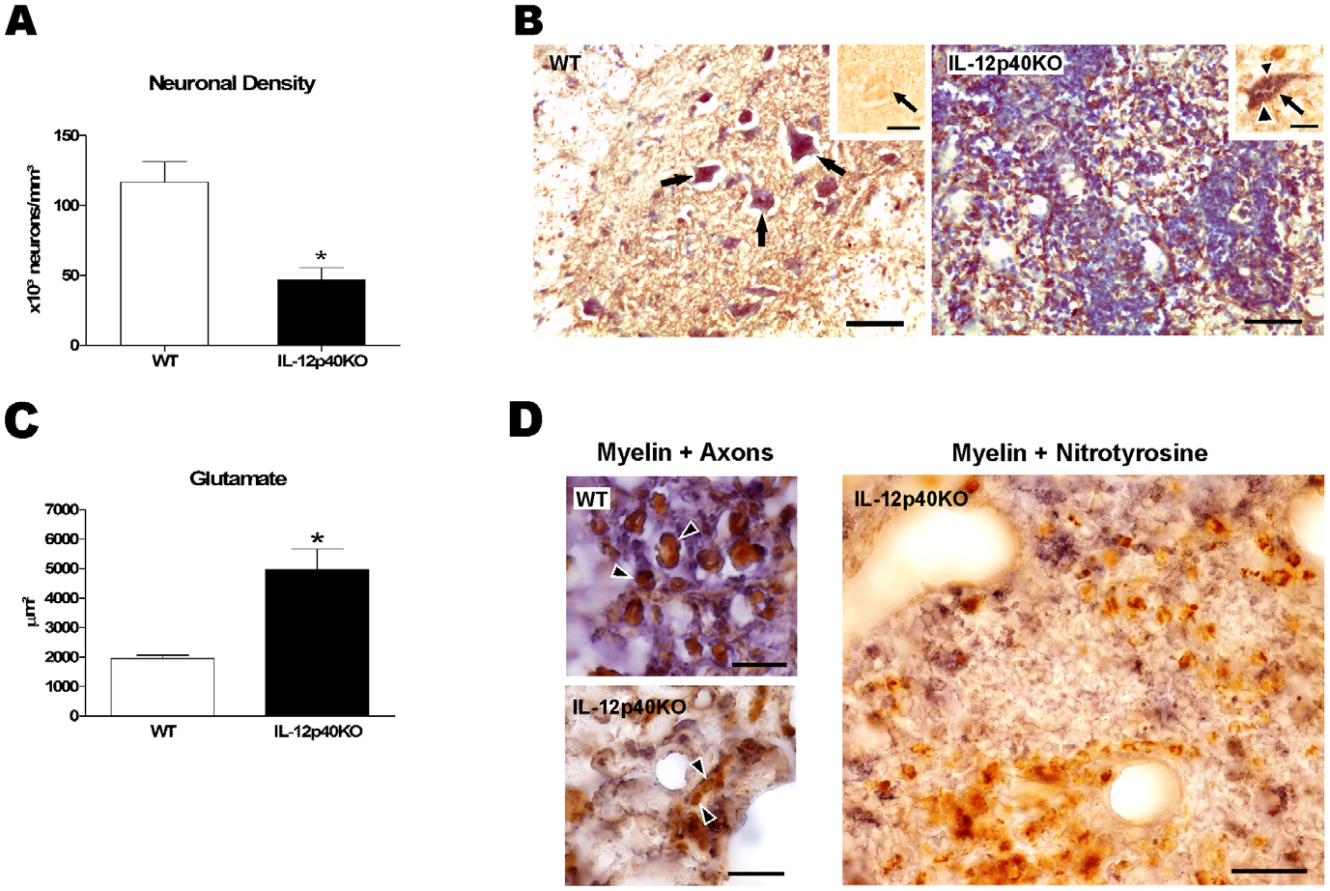
Neurodegenerative aspects of IL-12p40KO spinal cords. A) Paralyzed *T. cruzi*-infected IL-12p40KO mice exhibited a decreased neuronal density in the spinal cord, when compared to the WT counterparts. B) Intact WT spinal cord section (left) with healthy neurons (arrows) and a morphologically disrupted IL-12p40KO spinal cord section (right), which is rich in infiltrating cells. Sections were immunolabeled for NF-200 (a neuronal marker, in brown) and counterstained with Giemsa (for total nuclei, in blue). *T. cruzi* amastigote forms were not seen in the WT neurons (left panel inset, arrow points a neuron, in brown) but occurred in a few IL-12p40KO neurons (right panel inset, arrow points a neuron, in brown, and the arrowheads point amastigotes, in purple). Scale bars: left and right pictures, 50 µm; insets, 20 µm. C) Glutamate immunolabeling area was higher in the spinal cords of paralyzed *T. cruzi*-infected IL-12p40KO mice than in the paired WT ones. D) Myelin sheaths remained intact in infected WT spinal cords in the end-point of the infection (upper left picture, arrow-heads show myelin wrapped axons), while they were disrupted in the IL-12p40KO damaged tissue (lower left picture, arrow-heads show myelin-free axons). In the right picture, note that in the presence of nitrotyrosine (brown), little myelin debris (blue) is observed in the damaged IL-12p40KO tissue. Sections were double-immunolabeled to myelin (purple-blue) and neuronal soma and axons (brown, left pictures) or nitrotyrosine (brown, right picture). Scale bars: left smaller pictures, 10 µm; right picture, 100 µm. Data on both graphs are presented as the mean ± SEM. *p<0.05, according to the unpaired *t*-test.

We have observed that spinal cord neurons were affected in terminally-paralyzed infected IL-12p40KO mice ([11] and Figure 2A and B). According to the stereological analysis, the estimated neuronal density over the spinal cord decreased 60% (p<0.05) in the IL-12p40KO group (Figure 2A). Direct neuronal parasitism is an unlikely contributor to neuron death as we rarely found IL-12p40KO neurons infected with *T. cruzi* (Figure 2 B). When investigating the presence of potentially neurotoxic substances, we found an increased glutamate immunolabeled area over the entire spinal cord of the paralyzed IL-12p40KO mice (153.0%, p<0.05; Figure 2C). Moreover, double immunolabeling for simultaneous detection of nitrotyrosine (to indirectly visualize nitric oxide) and myelin basic protein revealed scarce myelin sheaths in areas that were strongly labeled to nitrotyrosine (Figure 2D).

**Figure 3:**
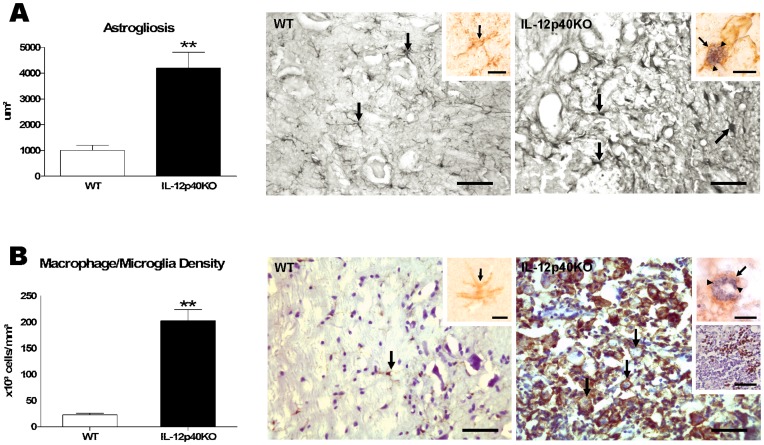
Astrogliosis and increased macrophage/microglia density in IL-12p40KO spinal cords. Paralyzed infected IL-12p40KO mice presented higher immunolabeled area to the astrocytic marker GFAP (A) and increased density of macrophages/microglia (B) in the spinal cord when compared to the matched infected WT group. Pictures in Figure A show astrocytes (arrows) in immunocompetent (left panel) and IL-12p40KO (right panel) spinal cords. No *T. cruzi*-infected astrocyte was found in WT tissue (left panel inset, arrow points an astrocyte), whereas in IL-12p40KO tissue, only a few astrocytes containing *T. cruzi* amastigotes were seen (right panel inset, arrow points an astrocyte and arrowheads point parasites). Pictures in Figure B show microglial cell (arrow) with long thin processes in WT tissue (left panel) and round-shaped phagocytes (arrows point macrophages or microglia) in a lesion area of an IL-12p40KO spinal cord section (right panel). While no parasite was found in the WT tissue (right panel inset, arrow shows a microglia), more than a third of all IL-12p40KO macrophages/microglias were infected (right panel upper inset, arrow shows a macrophage/microglia and arrowheads point parasites). M2 cells were visualized in the IL-12p40-KO spinal cord only (right panel lower inset, M2 cells in brown). Sections were immunolabeled to GFAP (A) and CD11b (B, also counterstained with Giemsa), GFAP and *T. cruzi* (A, inset), CD11b and *T. cruzi* (B, upper insets) or arginase I (B, right panel lower inset, also counterstained with hematoxylin). Scale bars: A and B, 50 µm; A insets and B upper insets, 20 µm; B right panel lower inset, 50 µm. Data on both graphs are presented as the mean ± SEM. **p<0.01, according to the unpaired *t*-test.

Reactive astrogliosis was analyzed via GFAP immunelabeling that revealed WT spinal cord gray and white matter astrocytes with small bodies and long thin processes (Figure 3A). IL-12p40KO spinal cords showed larger GFAP immunoreactive areas (317.0% increase, p<0.01) exhibiting activated astrocytes with larger cell bodies and hypertrophic cellular processes along the tissue, including the lesion areas (Figure 3A). Amastigote forms of *T. cruzi* were not detected in WT astrocytes but were occasionally found in the IL-12p40KO ones (Figure 3A, insets).

Few microglia cells were detected in the WT spinal cord, all of them displaying long thin processes (Figure 3B). In IL-12p40KO spinal cords, however, we observed abundant immunolabeled round-shaped macrophages/microglia. The majority of these activated cells were seen within lesions or in the surrounding areas (Figure 3B). In comparison to the WT group, IL-12p40KO mice exhibited a 780.6% (p<0.01) increase in the estimated macrophage/microglia density across the entire spinal cord (Figure 3B). Moreover, 36.3% (p<0.01) of all IL-12p40KO macrophages/microglia were infected with *T. cruzi* but no parasite amastigote nests were found in WT microglia (Figure 3B, upper insets). Of note, the arginase I immunolabeling revealed the presence of M2 cells in the IL-12p40-KO spinal cord only (Figure 3B, right panel lower inset). The fact that more than one-third of IL-12p40KO phagocytes were infected led us to investigate whether microglia cells from IL-12p40KO deficient mice dictate higher susceptibility to *T. cruzi* infection (Protocol S2). No difference in the infection ratio or in the parasite killing capacity was seen between paired WT and IL-12p40KO microglia cultures that were pre-treated or not with rIFN-γ (Figure S2).

These data suggests that WT mice mounted an efficient immune response in constraining the local parasitic load and preserved the spinal cord tissue while IL-12p40KO did not control the parasite dissemination and elicited a disproportionate inflammatory response.

### Spinal Cord Inflammation

**Figure 4:**
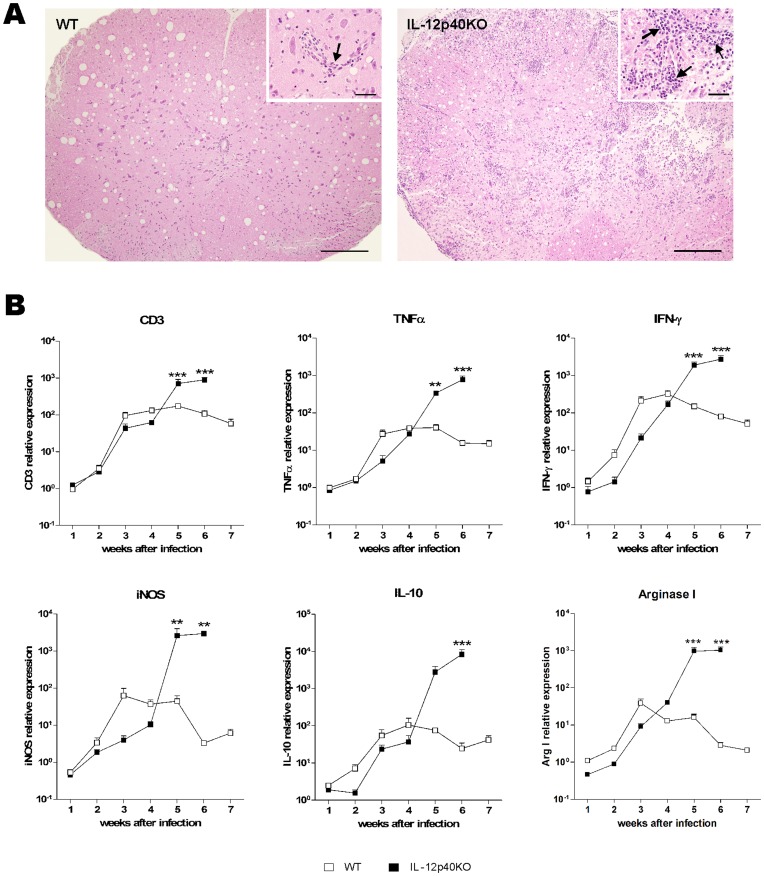
Spinal cord inflammation. A) After WT and IL-12p40KO mouse transcardiac perfusion, spinal cord upper lumbar intumescences were submitted to histopathological analysis. While WT spinal cord displayed preserved morphology (left picture), with few inflammatory cells (inset, arrow), IL-12p40KO spinal cord presented intense morphological disarrangement (right picture), due to the large amount of inflammatory foci (inset, arrows). Representative pictures of HE-stained spinal cord sections of WT and IL-12p40KO mice in the sixth week after infection. Scale bars: 100 µm; insets, 20 µm. B) Once a week during seven weeks, cDNA was obtained from the spinal cord lower lumbar segments of WT and IL-12p40KO mice and submitted to real time RT-PCR procedures, using primers for CD3, TNF-α, IFN-γ, iNOS, IL-10, arginase I and HPRT, β-actin and GAPDH as endogenous control. After the fourth week of infection, the transcription ratio of all genes analyzed tended to remission in the WT tissue, while they continued increasing in the IL-12p40KO spinal cord. Of note, also around the fourth week of infection, a switch in the transcription level of the analyzed genes was observed between the two mouse strains, when IL-12p40KO mice started expressing more RNA than WT ones. Mean ± SEM. **p<0.01; ***p<0.001 when comparing paired WT and IL-12p40KO groups according to Bonferroni post-tests. n = 4 for each strain in each time point.

Next we perform a kinetic analysis over seven weeks sampling the upper lumbar intumescences of WT and IL-12p40KO spinal cords. From the fourth week of infection on, the mononuclear infiltrating cells were increased in IL-12p40KO tissues (data not shown). Despite the small number of inflammatory foci, the spinal cord tissue remained preserved in WT mice (Figure 4A), while it displayed altered morphology in the IL-12p40KO group, especially in the sixth week after the infection (Figure 4A). Tracking the gene expression profile of target inflammatory genes, we observed that in WT spinal cords the amount of mRNA for CD3, TNF-α, IFN-γ, iNOS, IL-10 and arginase I increased up between the third and the fifth week after infection, decreasing thereafter (Figure 4B). In IL-12p40KO mice, the transcription ratio for these molecules showed a continuous ascending trend throughout the experimental time (Figure 4B). Of note, there is a stronger expression of these molecules in WT mice at the beginning of the infection denoting a faster response to the parasite. In fact, an approximate one-week delay in the transcription ratio for CD3, TNF-α, IFN-γ, iNOS, IL-10 and arginase I was observed in IL-12p40KO mice at the third and fourth weeks after infection. Interestingly, over expression for all transcripts occurred after the fourth week of infection (Figure 4B), correspondent to the period when IL-12p40KO mice started presenting motor impairments (Figure 1A). In additional experiments, we observed that neither IL-12p40KO, nor WT infected mice displayed local transcription of IL-17 (data not shown). Together, these data are consistent with a delayed and prolonged inflammatory response in the spinal cord of IL-12p40KO mice.

### Spinal Cord Parasitism

**Figure 5:**
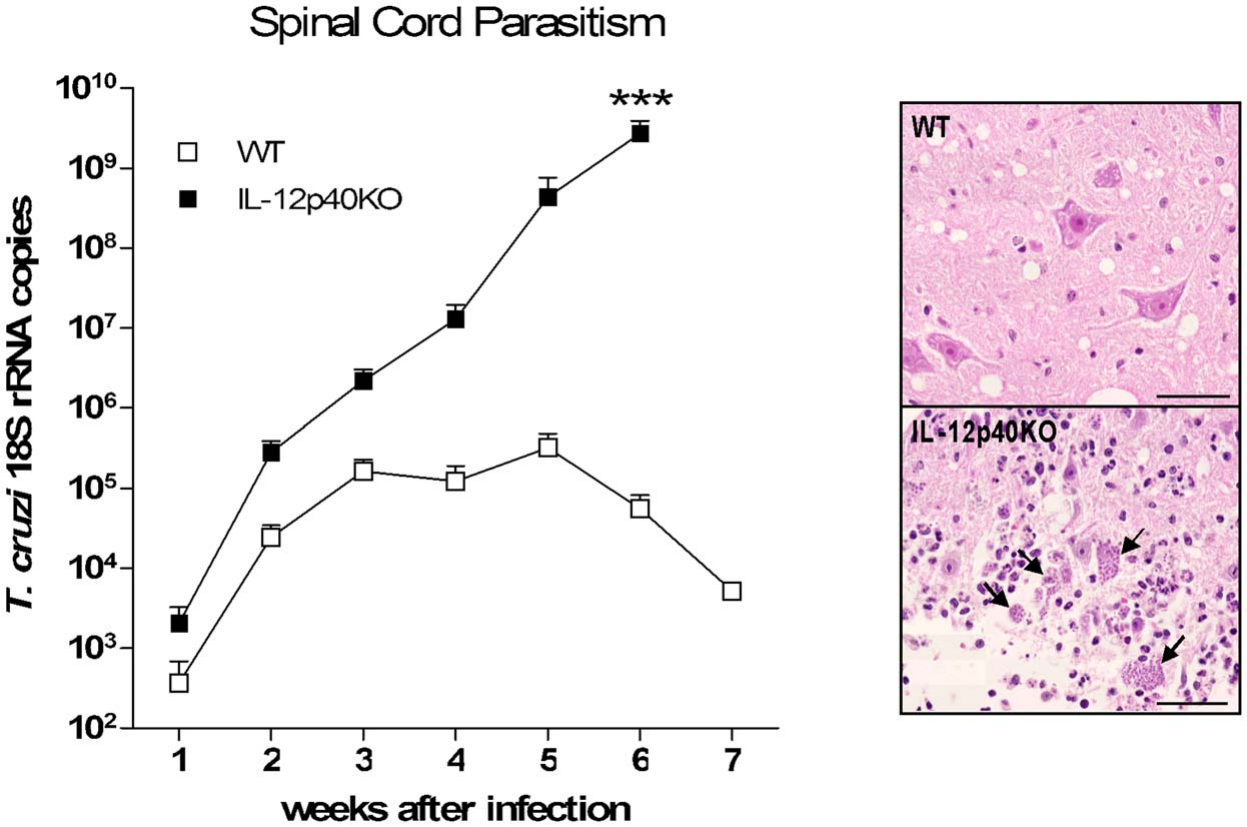
Spinal cord parasitism. After transcardiac perfusion, spinal cord lower lumbar segments were processed for RNA extraction and cDNA production. Real time RT-PCR was performed using primers for *T. cruzi* 18S rRNA and HPRT as endogenous control. For the absolute quantification, sample values were fitted into a standard curve, constructed from a pattern sample with known number of copies of *T. cruzi* 18S rRNA. IL-12p40KO mice presented an ascending parasitism while WT ones managed to constrain the infection by the third week, thus decreasing the parasitic load after the fifth week. Representative pictures of HE-stained upper lumbar spinal cord sections, obtained from WT (upper square) and IL-12p40KO mice (lower square) in the sixth week after the infection. Note the high amount of amastigote nests (arrows) in the IL-12p40KO tissue. Scale bars: 20 µm. Mean ± SEM. ***p<0.001 when comparing paired WT and IL-12p40KO groups according to Bonferroni post-tests. n = 4 for each strain in each time point.

In spite of the exacerbated inflammatory response represented by high local production and transcription of IFN-γ and nitric oxide related molecules relevant for *T. cruzi* killing [10], [25], the IL-12p40KO mice are not capable to control parasite dissemination in the spinal cord [11]. Nonetheless, when investigating the spinal cord parasitism by means of qRT-PCR, we surprisingly found *T. cruzi* 18S rRNA copies in WT early in the infection course (Figure 5). WT spinal cord parasitism persisted during all the experimental period, reaching its peak in the fifth week after the infection and decreasing thereafter (Figure 5). In IL-12p40KO spinal cord, parasitism was also detected by the first week after the infection, progressively increasing up to the end of the experimental period as expected (Figure 5). Of note, in the second week after the infection IL-12p40KO mice presented as much *T. cruzi* 18S transcripts as the maximum amount reached by WT mice in the fifth week after the infection (Figure 5).

Together this work implicates IL-12p40 as a key component for parasite control in the course of nervous tissue responses to *T. cruzi* infection strongly suggesting that an IL12/IL23-dependent signal is required early in infection to efficiently fight parasite expansion in the spinal cord and to preserve tissue integrity.

## Discussion

More than 100 years ago, Carlos Chagas carried out a detailed study, in which he described a new trypanosomiasis [26] caused by *T. cruzi* and further named as Chagas’ disease. In that study, Chagas reported the eventual compromise of the nervous system, especially in children. During many decades, much was done to better understand the disease, but little attention was given to the involvement of the nervous system, a field that remained unexplored for years. With the increasing co-occurrence of *T. cruzi* infection and immunossupression due to anti-allograft rejection drug treatments or HIV infection, cases of clinical CNS involvement in chagasic patients have been reported [7], [8], [27], [28], [29], [30].

Sylvio X10/4 is a low virulence clone of *T. cruzi* that does not cause patent parasitemia but induces chronic myocarditis with the characteristics of the human disease [31]. When infected by Sylvio X10/4 parasites, IL-12p40KO mice developed an ascending paralysis, from the tail to the forelimbs, prior to dying. These mice do not produce the cytokines IL-12 and IL-23, because the IL-12p40 subunit, shared by both interleukins, is not functional. In this way, due to the lack of IL-12 they fail to mount a full Th1 response, crucial for an efficient *T. cruzi* control [9], [10]. Moreover, in the absence of IL-23, they do not maintain the inflammatory Th17 lymphocytes [32], which produce IL-17, that was reported to be protective during the acute phase of experimental Chagas’ disease [33], [34]. Of note, the spontaneous motor activity herein reported, showing an ascending paralysis in Sylvio X10/4-infected IL-12p40KO mice was corroborated by the unbiased infrared motion sensor data and the inclined plane test, the latter indicating the decrease of limb muscular tonus, which was evident by the appearance of distal tail palsy days before forelimb paralysis.

The failure of IL-12p40KO mice to mediate the control of *T. cruzi* dissemination in the spinal cord seemed to be more related to a local defect on controlling tissue parasitism than a massive entrance of parasites, favored by a systemic incapacity on controlling *T. cruzi* dissemination. This interpretation is supported by our current data showing that in the second week after infection, when no parasite was seen in the blood stream, the amount of *T. cruzi* 18S rRNA transcripts in the spinal cord of knockout mice was ever as high as WT individuals would reach. This is important, because, conversely to IL-12p40KO mice, WT mice were capable to successfully control the spinal cord infection, which decreased in approximately one hundred times from the fifth to the seventh week after infection.

The proliferation of astrocytes and microglia is a common feature of the damaged CNS tissue [35], [36], that aims to reestablish local homeostasis. Considered resident macrophages of the CNS, microglia when activated releases substances that are neurotoxic in high concentrations, such as free radicals, nitric oxide, proteases and cytokines [12], [15], [37]. Notwithstanding, an increase number of studies have demonstrated that astrocytes also perform immune functions [13], as the production of pro-inflammatory cytokines and chemokines [13], [38]. As a response to the colonization of the IL-12p40-KO spinal cord by *T. cruzi*, we observed in the phase of established motor impairments an increased immunoreactive area for the astrocytic marker GFAP, as well as an increased density of CD11b^+^ phagocytes, correspondent to microglia and infiltrating macrophages.

If in one hand inflammation is benefic for reestablishing tissue homeostasis under injuries, on the other hand it may become harmful when the antigenic stimulus persists, evoking the continuous release of potential cytotoxic substances. In this context, high concentrations of TNF-α and NO have been shown to impair glutamate uptake by astrocytes [39], [40] intensifying neurodegeneration and contributing to the death of oligodendrocytes, thus causing demyelinization [41], [42], [43], [44]. According to our results, for WT and IL-12p40KO mouse lineages, the transcription of pro-inflammatory substances and *T. cruzi* parasitism in the spinal cord evolved in parallel. Nevertheless, while WT mice managed to constrain the infection in the spinal cord and thus to diminish the loading of pro-inflammatory mediators, IL-12p40KO mice presented an ascendant *T. cruzi* parasitism, accompanied by intense local inflammation. Curiously, the curves for CD3, TNF-α, IFN-γ and iNOS transcripts of the two mouse strains intersected immediately after the fourth week of infection, a time point that is also marked by the appearance of motor impairments in IL-12p40KO mice, suggesting that the crescent inflammation thereafter presented by these mice is related to the development of the neurodegenerative process [11], which minimally results from the death of infected neurons. Of note, the increased gene expression of the anti-inflammatory molecules IL-10 and arginase I, the late one involved in macrophages differentiation to the less aggressive M2 profile [45], could be seem as a protective mechanism that aims to slow down the inflammation, but fails due to the parasite persistence.

Previously, we reported that in the phase of complete motor paralysis the amount of IFN-γ and nitrated proteins in the spinal cord of *T. cruzi*-infected IL-12p40KO mice was notably higher than in infected WT mice [11]. Additionally, in the present study we found parasites along the entire knockout spinal cord, being microglia and infiltrating macrophages the main targets of infection. At first glance, a deficit in the killing capacity of microglia/macrophages is somewhat surprising if we consider the high levels of IFN-γ that are locally produced in the spinal cords of IL-12p40KO mice. A candidate hypothesis to explain why IFN-γ exposure insufficiently activates microglia/macrophages for efficient local *T. cruzi* control could be a deficit in the priming of these phagocytes [46], consequent to the low background levels of IFN-γ in non-infected IL-12p40KO mice [47]. In this context, adult IL-12p40KO spinal cord macrophages/microglia would be less responsive to the IFN-γ produced after the infection than the WT cells, as reported to IL-12p40KO mouse peritoneal macrophages which kill less *T. cruzi* parasites than the WT ones under the same *in vitro* rIFN-γ treatment [48]. Yet, according to our results, WT and IL-12p40-KO microglia behaved the same way when stimulated with the same amount of rIFN-γ previously to *T. cruzi* infection, most probably due to the lack of priming of both cell cultures, which were obtained from newborn mice.

The importance of IFN-γ in Chagas’ disease is highlighted by the fact that infected IFN-γKO mice, or mice treated with anti-IFN-γ antibodies, present high parasitemia and high mortality early in the infection, accompanied by increased tissue parasitism [10], [49]. According to our data, both WT and IL-12p40KO mice locally transcribed IFN-γ mRNA in all time points. This could be because in spite that IL-12 is important for Th1 polarization, IFN-γ production is not totally dependent on the presence of this cytokine. In this way, IL-18 was reported to synergize with IL-12 stimulating IFN-γ production [50], or to induce IFN-γ production even alone, as demonstrated for *T. cruzi*-infected IL-12p35KO mice which CD4^+^ T cells managed to produce IFN-γ in a IL-18 dependent way [51]. Of note, both astrocyte and microglia cell populations were shown to produce IL-18 [52], [53]. Not surprising, Sylvio X10/4-infected IL-12p40KO mice managed to transcribe high levels of mRNA for IFN-γ late in the infection. Despite it, these mice did not managed to control the local parasitism. According to our data, the optimum transcription level for IFN-γ is achieved by WT mice around the third week after infection, when they start to control the parasite in the spinal cord. Meanwhile, at this time point, the IFN-γ transcription presented by IL-12p40KO mice is much lower, equalizing to WT mice only one week after (around the fourth week), when the local parasitism is much higher in knockout than in WT mice. Thus, even being produced in considerable amounts, the levels of IFN-γ in the knockout tissue would never be sufficient to constrain the infection due to the parasite burst.

In view of our data, we suggest that a delay in the early production of IFN-γ in the spinal cord of *T. cruzi*-infected IL-12p40KO mice is responsible for the late or inefficient activation of microglia and infiltrating macrophages, thus favoring the uncontrolled dissemination of the protozoan through the nervous tissue. Consequently, phagocytes and astrocytic populations become augmented in an effort to constrain the infection. Nevertheless, the lack of an efficient immune response and the persistence of the protozoan infection favor the continuous release of substances that become neurotoxic at high levels, such as NO and glutamate. Finally, as a result of the neurodegenerative process, motor dysfunction is established.

## Supporting Information

Figure S1 Automatic recording of motor activity. The unbiased automatic recording of motor activity was performed for *T. cruzi*-infected WT (n = 6) and IL-12p40KO (n = 8) mice, where they were individually placed inside polyethylene cages that were equipped with an infrared motion sensor so as to register motor activity. The parameters evaluated were the number (A) and duration (B) of periods that mice stayed at rest and the number (C) and duration (D) of large movements. The strains behaved differently regarding both the number (p<0.05) and duration (p<0.01) of resting stops and the number (p<0.01) and duration (p<0.01) of large movements (C and D). Mean ± SEM. Variation between strains was analyzed by 2-way ANOVA.
(TIF)

Figure S2 Microglia infectiveness and parasitic killing capacity. WT and IL-12p40KO newborn mouse microglias, untreated or treated with rIFN-γ (100 pg/mL) were infected with *T. cruzi* (1∶1 ratio) and analyzed for the presence of intracellular parasites. A) No difference in the percentage of infected cells was seen between paired WT and IL-12p40KO cultures submitted to the same treatment. Nevertheless, there was a decrease in the infection rate of each group when cells were treated with 100 pg/mL rIFN-γ (p<0.001 for both cell strains). B) With regard to the number of *T. cruzi* amastigote forms per infected cell, there was no difference between paired WT and IL-12p40KO cultures under the same treatment. Nevertheless, when cells of the same lineage were stimulated with 100 pg/mL rIFN-γ, the number of parasite proliferative forms diminished in comparison to untreated cells (p<0.001 for both strains). Mean ± SEM. Data are expressed as an average of three independent experiments, performed in triplicate.
(TIF)

Protocol S1 Behavioral evaluation. The automatic recording of motor activity based on movement time [1], without any intervention of the investigator, was assessed using an infrared motion sensor monitor (Coulbourn Instruments). Each mouse was individually placed in a polyethylene cage (37×17×30 cm) that was equipped with an infrared motion sensor and monitored for 30 min. The data corresponded to the number of events and to the total time during which the animal remained stopped or moved over a period of 0.01 s (resting events and resting time) or during which the animal constantly moved for a period higher than 1.0 s (large-movement events and large-movement time). 1. Andrade MS, Mendonca LM, Chadi G (2010) Treadmill running protects spinal cord contusion from secondary degeneration. Brain Res 1346∶266–278.
(DOC)

Protocol S2 Microglial cell culture and infection by *T. cruzi*. Primary glial cell culture and microglial isolation were done according to Neumann *et al.* [2], with minor modifications. In brief, brains of postnatal day 3 to 5 WT and IL-12p40KO mice were dissociated by trituration after mechanical removal of the meninges. Cells were cultured in medium (DMEM-F12, Gibco) containing 10% FCS (Invitrogen) and 1% penicillin/streptomycin (Gibco) during 14 days, to form a glial monolayer. Cultures were shacked on a rotary shaker (200 rpm, 2 h) and the microglial cell-enriched supernatant was collected. Cells were harvested in eight-chamber slides (Lab-Tek, 50×10^3^ cells/well) for 1 h, and then the non-adherent cells were removed. Purity of isolated microglia was higher than 95%, as confirmed by flow cytometry with FITC-labeled antibody against CD11b (BD Biosciences). After isolation, seeded cells were treated or not with rIFN-γ (100 pg/mL, Pharmingen) in medium for 48 h. Cells were washed 3× PBS 0.1 M and then infected with *T. cruzi* Sylvio X10/4, obtained as described above, in a 1∶1 ratio (parasite : microglia) for 2 h. Cultures were washed again 3× PBS 0.1 M to remove extracellular parasites and left in medium for 48 h. Cells were fixed in paraformaldehyde 4% for 30 min, and stained with HE. Under light microscopy (1000× magnitude), 100 cells per chamber were analyzed and the number of amastigote forms of *T. cruzi* per infected cell was registered, as so it was possible to calculate the percentage of infected cells as well as the number of amastigote forms per infected cell. Experiments were done in triplicates. 2. Neumann H, Misgeld T, Matsumuro K, Wekerle H (1998) Neurotrophins inhibit major histocompatibility class II inducibility of microglia: involvement of the p75 neurotrophin receptor. Proc Natl Acad Sci U S A 95∶5779–5784.
(DOC)
