# Supplementary material for: IL-12p40 Deficiency Leads to Uncontrolled Trypanosoma cruzi Dissemination in the Spinal Cord Resulting in Neuronal Death and Motor Dysfunction
Source: PLoS One. 2012 Nov 12;7(11):e49022. doi: 10.1371/journal.pone.0049022 (PMC3495776; doi:10.1371/journal.pone.0049022)
Supplement: Protocol S1 — Behavioral evaluation. The automatic recording of motor activity based on movement time [1], without any intervention of the investigator, was assessed using an infrared motion sensor monitor (Coulbourn Instruments). Each mouse was individually placed in a polyethylene cage (37×17×30 cm) that was equipped with an infrared motion sensor and monitored for 30 min. The data corresponded to the number of events and to the total time during which the animal remained stopped or moved over a period of 0.01 s (resting events and resting time) or during which the animal constantly moved for a period higher than 1.0 s (large-movement events and large-movement time). 1. Andrade MS, Mendonca LM, Chadi G (2010) Treadmill running protects spinal cord contusion from secondary degeneration. Brain Res 1346∶266–278. (DOC) [file pone.0049022.s003.doc]

**Protocol S1. Behavioral evaluation.** The automatic recording of motor activity based on movement time [1], without any intervention of the investigator, was assessed using an infrared motion sensor monitor (Coulbourn Instruments). Each mouse was individually placed in a polyethylene cage (37x17x30 cm) that was equipped with an infrared motion sensor and monitored for 30 min. The data corresponded to the number of events and to the total time during which the animal remained stopped or moved over a period of 0.01 s (resting events and resting time) or during which the animal constantly moved for a period higher than 1.0 s (large-movement events and large-movement time).

1. Andrade MS, Mendonca LM, Chadi G (2010) Treadmill running protects spinal cord contusion from secondary degeneration. Brain Res 1346: 266-278.
