# Supplementary material for: IL-12p40 Deficiency Leads to Uncontrolled Trypanosoma cruzi Dissemination in the Spinal Cord Resulting in Neuronal Death and Motor Dysfunction
Source: PLoS One. 2012 Nov 12;7(11):e49022. doi: 10.1371/journal.pone.0049022 (PMC3495776; doi:10.1371/journal.pone.0049022)
Supplement: Protocol S2 — Microglial cell culture and infection by T. cruzi. Primary glial cell culture and microglial isolation were done according to Neumann et al. [2], with minor modifications. In brief, brains of postnatal day 3 to 5 WT and IL-12p40KO mice were dissociated by trituration after mechanical removal of the meninges. Cells were cultured in medium (DMEM-F12, Gibco) containing 10% FCS (Invitrogen) and 1% penicillin/streptomycin (Gibco) during 14 days, to form a glial monolayer. Cultures were shacked on a rotary shaker (200 rpm, 2 h) and the microglial cell-enriched supernatant was collected. Cells were harvested in eight-chamber slides (Lab-Tek, 50×103 cells/well) for 1 h, and then the non-adherent cells were removed. Purity of isolated microglia was higher than 95%, as confirmed by flow cytometry with FITC-labeled antibody against CD11b (BD Biosciences). After isolation, seeded cells were treated or not with rIFN-γ (100 pg/mL, Pharmingen) in medium for 48 h. Cells were washed 3× PBS 0.1 M and then infected with T. cruzi Sylvio X10/4, obtained as described above, in a 1∶1 ratio (parasite : microglia) for 2 h. Cultures were washed again 3× PBS 0.1 M to remove extracellular parasites and left in medium for 48 h. Cells were fixed in paraformaldehyde 4% for 30 min, and stained with HE. Under light microscopy (1000× magnitude), 100 cells per chamber were analyzed and the number of amastigote forms of T. cruzi per infected cell was registered, as so it was possible to calculate the percentage of infected cells as well as the number of amastigote forms per infected cell. Experiments were done in triplicates. 2. Neumann H, Misgeld T, Matsumuro K, Wekerle H (1998) Neurotrophins inhibit major histocompatibility class II inducibility of microglia: involvement of the p75 neurotrophin receptor. Proc Natl Acad Sci U S A 95∶5779–5784. (DOC) [file pone.0049022.s004.doc]

**Protocol S2. Microglial cell culture and infection by *T. cruzi*.** Primary glial cell culture and microglial isolation were done according to Neumann *et al.* [1], with minor modifications. In brief, brains of postnatal day 3 to 5 WT and IL-12p40KO mice were dissociated by trituration after mechanical removal of the meninges. Cells were cultured in medium (DMEM-F12, Gibco) containing 10% FCS (Invitrogen) and 1% penicillin/streptomycin (Gibco) during 14 days, to form a glial monolayer. Cultures were shacked on a rotary shaker (200rpm, 2h) and the microglial cell-enriched supernatant was collected. Cells were harvested in eight-chamber slides (Lab-Tek, 50x10³ cells/well) for 1h, and then the non-adherent cells were removed. Purity of isolated microglia was higher than 95%, as confirmed by flow cytometry with FITC-labeled antibody against CD11b (BD Biosciences). After isolation, seeded cells were treated or not with rIFN-γ (100pg/mL, Pharmingen) in medium for 48h. Cells were washed 3x PBS 0.1M and then infected with *T. cruzi* Sylvio X10/4, obtained as described above, in a 1:1 ratio (parasite : microglia) for 2h. Cultures were washed again 3x PBS 0.1M to remove extracellular parasites and left in medium for 48h. Cells were fixed in paraformaldehyde 4% for 30min, and stained with HE. Under light microscopy (1000x magnitude), 100 cells per chamber were analyzed and the number of amastigote forms of *T. cruzi* per infected cell was registered, as so it was possible to calculate the percentage of infected cells as well as the number of amastigote forms per infected cell. Experiments were done in triplicates.

1. Neumann H, Misgeld T, Matsumuro K, Wekerle H (1998) Neurotrophins inhibit major histocompatibility class II inducibility of microglia: involvement of the p75 neurotrophin receptor. Proc Natl Acad Sci U S A 95: 5779-5784.
